# Extraintestinal Helminth Infection Limits Pathology and Proinflammatory Cytokine Expression during DSS-Induced Ulcerative Colitis: A Role for Alternatively Activated Macrophages and Prostaglandins

**DOI:** 10.1155/2015/563425

**Published:** 2015-05-18

**Authors:** Yadira Ledesma-Soto, Blanca E. Callejas, César A. Terrazas, Jose L. Reyes, Arlett Espinoza-Jiménez, Marisol I. González, Sonia León-Cabrera, Rosario Morales, Jonadab E. Olguín, Rafael Saavedra, Steve Oghumu, Abhay R. Satoskar, Luis I. Terrazas

**Affiliations:** ^1^Unidad de Biomedicina, Facultad de Estudios Superiores Iztacala, Universidad Nacional Autónoma de México, Avenida De Los Barrios 1, Los Reyes Iztacala, 54090 Tlalnepantla, MEX, Mexico; ^2^Department of Pathology, Medical Center, The Ohio State University, Columbus, OH 43221, USA; ^3^Department of Physiology and Pharmacology, Faculty of Medicine, University of Calgary, Calgary, AB, Canada T2N 4N1; ^4^Departamento de Inmunología, Instituto de Investigaciones Biomédicas, UNAM, 04510 Mexico, DF, Mexico

## Abstract

Chronic inflammation of the intestinal mucosa is characteristic of inflammatory bowel diseases such as ulcerative colitis and Crohn's disease. Helminth parasites have developed immunomodulatory strategies that may impact the outcome of several inflammatory diseases. Therefore, we investigated whether *Taenia crassiceps* infection is able to decrease the inflammatory effects of dextran sulfate sodium- (DSS-) induced ulcerative colitis in BALB/c and C57BL/6 mice. Preinfection significantly reduced the manifestations of DSS-induced colitis, as weight loss and shortened colon length, and decreased the disease activity index independently of the genetic background of the mice. *Taenia* infection decreased systemic levels of proinflammatory cytokines while increasing levels of IL-4 and IL-10, and the inflammatory infiltrate into the colon was also markedly reduced. RT-PCR assays from colon showed that *T. crassiceps*-infected mice displayed increased expression of Arginase-1 but decreased expression of iNOS compared to DSS-treated uninfected mice. The percentages of T regulatory cells were not increased. The adoptive transfer of alternatively activated macrophages (AAM*Ф*s) from infected mice into mice with DSS-induced colitis reduced the severity of colon inflammation. Administration of indomethacin abrogated the anticolitic effect of *Taenia*. Thus, *T. crassiceps *infection limits the pathology of ulcerative colitis by suppressing inflammatory responses mechanistically associated with AAM*Ф*s and prostaglandins.

## 1. Introduction

Helminth parasites have developed complex strategies to modulate the immune responses of their hosts through utilizing versatile immunoregulatory mechanisms to avoid immune effector cells and molecules. These parasites bias the immune response toward Th2 and/or a regulatory environment associated with high levels of IL-4, IL-13, IL-9, IL-5, and IL-10; also, infection with helminths compromises immunity to other unrelated infections and may also affect the efficacy of vaccines [1]. Various cell populations are affected by helminth infections, including macrophages, dendritic cells (DCs), T regulatory cells (Treg), mast cells, and neutrophils [2]. Thus, helminths use multiple means to escape or modulate the immune response in their hosts [3]. A growing body of evidence in recent years has shown that immunomodulatory activities displayed by helminths can impact in different ways the outcomes of several inflammatory diseases, including multiple sclerosis (MS), arthritis, type 1 diabetes (T1D), and inflammatory bowel diseases (IBD) such as ulcerative colitis and Crohn's disease [4].

One inflammatory disease of alarming frequency is IBD. Although these diseases were considered rare 50 years ago, today some developed countries report 1 patient for every 250 people [5]. In Europe up to 0.3% of the population suffer from IBD [6]. An association has been suggested between an absence of helminth infection and the increase in cases of IBD [4]. Rodent models of IBD have been used to study the mechanisms underlying the development of these inflammatory diseases [7], and several experimental studies support the idea that IBD (mainly ulcerative colitis) can be regulated by helminth infection [8, 9]. Human trials have demonstrated some efficacy for patients but with undesirable side effects [10–12]. Nevertheless, seven different species of helminths, mainly gastrointestinal ones, have been tested for their role in modulating the development of ulcerative colitis, some with adverse effects [10, 11, 13–15].

*Taenia crassiceps* (class* Cestoda*) is a helminth parasite that can be found in its adult form in the small intestine of canids and in its larval stage (metacestode) in the muscles and peritoneal and pleural cavities of rodents. An interesting feature of* T. crassiceps* is its ability to reproduce asexually through budding at the larval stage. This characteristic permits the parasite to remain in and colonize its hosts for long periods of time; thus, 6–8 weeks after the intraperitoneal (i.p.) inoculation of 10–20 metacestodes, hosts can harbor hundreds of parasites. In addition, the parasite in its larval stage is innocuous to humans, is macroscopic in size, does not kill the host, and is able to cause chronic infection with a minimum amount of damage in mice. We found an inhibition of proinflammatory responses, induction of Th2-biased immune responses, myeloid-derived suppressor cells, impairment of DC maturation, and lymphocyte proliferative responses, as well as recruitment of alternatively activated macrophages (AAM*Ф*s) during* T. crassiceps* infection, reviewed in [16]. Such immunoregulatory properties of this helminth had important beneficial effects on the development of experimental autoimmune encephalomyelitis (EAE, a murine model for multiple sclerosis) and T1D without side effects on the hosts [17, 18], whereas no effect at all was observed on arthritis [19].

Most of the effects of helminths on colitis have been studied using gastrointestinal infections, and consequently the impact of infection with helminths on organs outside the parasite's location has received much less attention [20]. For this reason and given the anti-inflammatory and immunoregulatory mechanisms of* T. crassiceps* infection, in this work we evaluated the effect of an extraintestinal infection on the development of dextran sulfate sodium- (DSS-) induced colitis.

## 2. Material and Methods

### 2.1. Mice

Female BALB/c or C57BL/6 mice 6–8 weeks of age were purchased from Harlan Laboratories (México) for use in some experiments. Mice were maintained in a pathogen-free environment at the FES-Iztacala, UNAM, animal facility according to Faculty Animal Care and Use Committee and government guidelines (official Mexican regulation NOM-062-ZOO-1999), which are in strict accordance with recommendations in the Guide for the Care and Use of Laboratory Animals of the National Institutes of Health (USA). Mice were sacrificed using a CO_2_ chamber, and all efforts were made to minimize pain. In some experiments C.Cg-*Foxp3* ^*tm1Tch*^/J reporter mice (Jackson Labs, USA) were used to detect the presence of T regulatory cells.

### 2.2. Parasites and Infection

Metacestodes of* T. crassiceps* were harvested from the peritoneal cavities of female BALB/c mice after 2–4 months of infection. The cysticerci were washed four times in sterile phosphate-buffered saline (PBS; 0.15 M, pH 7.2). Experimental infection was achieved via i.p. injection of 20 small (approximately 2 mm in diameter) nonbudding cysticerci of* T. crassiceps* suspended in 0.3 mL PBS per mouse (BALB/c). Because C57BL/6 mice are resistant to low doses (10–20 metacestodes) of* T. crassiceps*, we infected all C57BL/6 mice with 40 metacestodes.

### 2.3. Development and Assessment of DSS-Induced Colitis

DSS (MW: 35,000–50,000; MP Biomedicals, Solon, OH, USA) was administered ad libitum dissolved at 4% in drinking water for 7 to 10 days.

### 2.4. Assessment of Disease Activity Index (DAI) Score and Weight Loss

To assess the severity of colitis, we monitored DAI scores and weight loss daily. The DAI score was calculated as the sum of the diarrheal score and the bloody stool score as follows: 0 = normal stool and normal-colored stool, 1 = mildly soft stool and brown stool, 2 = very soft stool and reddish stool, and 3 = watery stool and bloody stool.

### 2.5. Histology

Colon tissue samples were fixed in 10% formalin and embedded in paraffin. Then 5 micrometer-thick tissue sections were prepared and stained with hematoxylin and eosin (HE) to evaluate mucosal damage. The sections were also stained with Alcian blue to evaluate the presence of goblet cells. We calculated the number of Alcian blue-positive goblet cells per five power fields (40x) using an Axio Vert.A1 microscopy (Carl Zeiss, Gottingen, Germany).

### 2.6. Cytokine Measurement

IL-4, IL-10, IL-17A, IL-17E, and TNF-*α* levels were quantified in mouse serum at the indicated times. Kits were used according to the manufacturer's instructions (Peprotech México, Mexico City, Mexico, and Biolegend, San Diego, CA, USA, for IL-17E).

### 2.7. Peritoneal Macrophage Purification, Adoptive Transfer, and In Vitro Suppression

Peritoneal exudate cells were isolated from the peritoneal cavities of* T. crassiceps-*infected mice 8 weeks after infection. Fc receptors were blocked by incubating the cells with mouse serum for 10 min at 4°C. Then cells were labeled with anti-F4/80 APC and anti-mannose receptor (FITC; 0.25 ug/10^6^ cells; Biolegend) for 20 min at 4°C. F480^+^MR^+^ and F480^+^MR^−^ populations were high-speed-sorted using a FACs Aria III flow cytometer. The viability of the cells was 90%, and purity was 95%. One million purified F4/80^+^MR^+^ or F4/80^+^MR^−^ cells were injected intraperitoneally into BALB/c mice 2 days after the start of DSS treatment. In addition, the antiproliferative properties of sorted F4/80^+^MR^+^ or F4/80^+^MR^−^ cells on T cells were tested. Briefly, total CFSE-labeled splenocytes from naïve mice were plated in 96-well plates previously coated with anti-CD3/CD28 (2 *µ*g/mL). After 4 h, F4/80^+^MR^+^ or F4/80^+^MR^−^ cells were added in different ratios. Proliferation was evaluated after 72 h on CD8- or CD4-gated populations by CFSE dilution assay using a FACSCalibur cytometer.

### 2.8. Flow Cytometry Analysis of Monocytes and T Regulatory Cells

Single-cell suspensions of circulation and lamina propria obtained during the sacrifice were stained with specific antibodies against CD11b (M1/70), Ly6C (HK1.4), Ly6G (1A8; all from Biolegend), and CCR2 (R&D Systems, USA) for 30 min at room temperature. To isolate colonic lamina propria cells, we flushed colons of their luminal content with cold PBS, opened them longitudinally, and cut them into 0.5 cm pieces. Epithelial cells and mucus were removed via 30 min incubation with HBSS containing 2% FBS, 2 mM EDTA, at 37°C and shaking at 50 g. Colon pieces were digested in DMEM containing 2 mg/mL Collagenase VIII (Sigma) and 40 *µ*g/mL DNase I (Invitrogen) for 2 h at 37°C with shaking at 250 rpm. The digested cell suspension was then washed with DMEM with 10% FBS, passed sequentially through 100 and 40 *µ*m cell strainers, and pelleted by centrifugation at 448 g for 10 min. Cells were subsequently separated by centrifugation through Percoll. Finally, cells from the same groups were pooled for analysis. Analyses of cells were performed using the FACSCalibur system and Cell Quest software (Becton Dickinson).

For the analysis of T regulatory cells, single spleen cells and PECs suspensions were obtained from C.Cg-*Foxp3* ^*tm1Tch*^/J reporter mice and stained with CD4 and CD25 (Biolegend) and gated on the CD4^+^ cell population. From this population CD25 and FOXP3 expression were analyzed.

### 2.9. In Vivo Suppression of Prostaglandin E_2_

To block the production of prostaglandin E_2_ in mice, we daily subjected mice to i.p. injection with indomethacin (3 mg per kg body weight). Controls were injected with DMSO 0.5% in bicarbonate buffer 5% as a vehicle control.

### 2.10. Statistical Analysis

Data were analyzed either by one-way analysis of variance followed by Tukey's multiple comparisons test or by unpaired two-tailed *t*-tests with GraphPad Prism 5 (San Diego, CA, USA).

## 3. Results

### 3.1. *Taenia crassiceps* Infection Decreases the Severity of DSS-Induced Colitis Independent of Genetic Background

**Figure 1:**
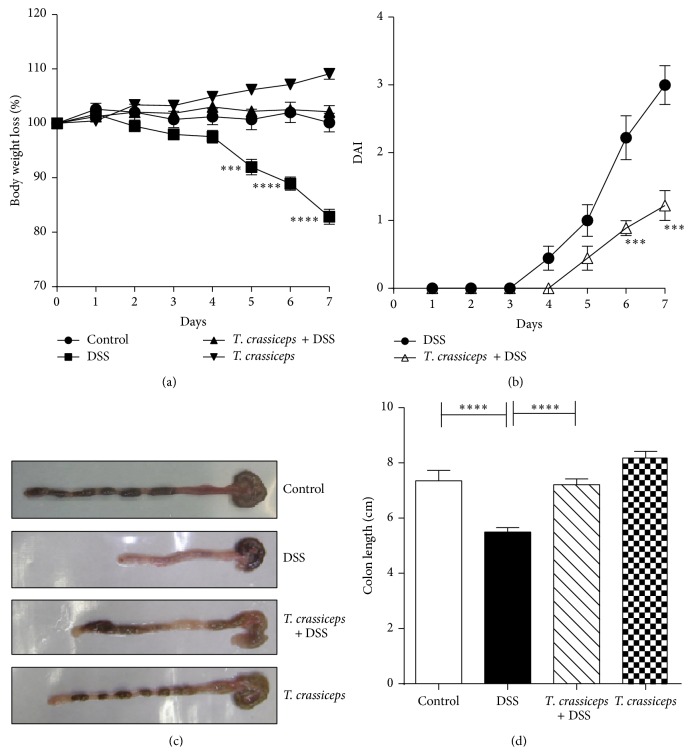
*T. crassiceps-*infected mice efficiently control colitis-associated pathology. Course of ulcerative colitis in* T. crassiceps*-infected and uninfected mice following 7 days of treatment with DSS at 4%. (a) Body weight change. (b) Clinical score. (c) Photograph of gross pathology of colons from different groups of mice. (d) Length of colon of infected and uninfected mice with ulcerative colitis. Bars represent the mean ± SD for six mice per group. ^*^ *P* < 0.05, ^***^ *P* < 0.003. All data are representative of three independent experiments.

We found that i.p. injection with the cestode* T. crassiceps* induces immunomodulation in its hosts. To formally assess the possible role of this extraintestinal helminth infection in the modulation of inflammatory responses in the colon, we explored whether the presence of this parasite in the peritoneal cavity of the host would modulate the severity of disease in an experimental model of ulcerative colitis. BALB/c and C57BL/6 mice previously infected (6 weeks) or not with* T. crassiceps* were exposed to DSS 4% or 3%, respectively, in drinking water for 7–9 days. The DAI was assessed daily as an average of loss of body weight and signs of rectal bleeding and diarrhea. Under such experimental conditions* T. crassiceps*-infected mice of both strains did not lose weight, whereas uninfected mice had significant progressive weight loss over time, weighing up to 20% of their initial body weight less at day 7 of exposure to DSS (Figure 1(a) for BALB/c; data not shown for C57BL/6). In line with these observations, DSS-treated* T. crassiceps*-infected mice developed reduced signs of morbidity (DAI) over the course of the disease compared to uninfected mice similarly treated with DSS (Figure 1(b)). Consistent with this, at necropsy, reduced colon shortening was found in* T. crassiceps*-infected mice after exposure to DSS compared to uninfected mice; this observation was similar for both strains of mice (Figures 1(c)-1(d) for BALB/c and Figure 4(a) for C57BL/6).

**Figure 2:**
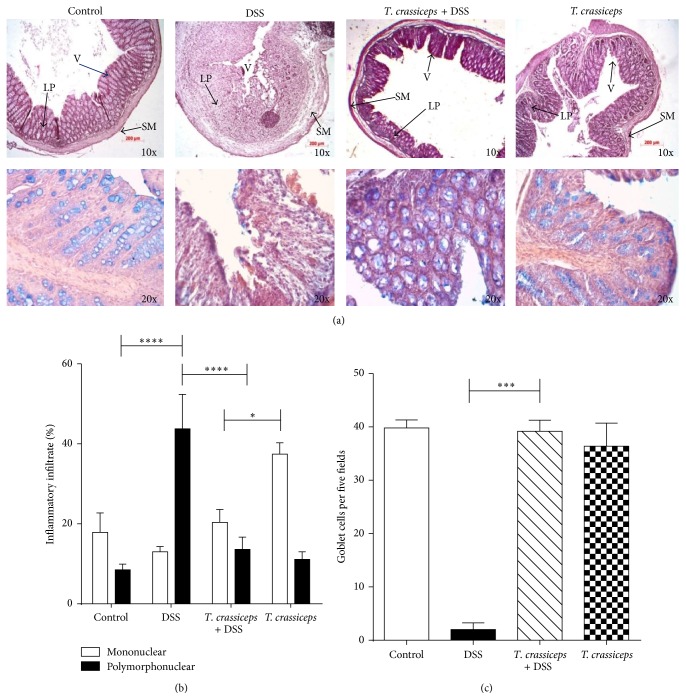
*T. crassiceps*-infected mice do not display severe pathology during ulcerative colitis. (a) Upper panel, colon tissue histology stained with H&E and showing colonic inflammation in different groups: magnification is 10x; bottom panel, Alcian blue-stained goblet cells (blue): magnification is 20x. (b) Percentages of neutrophils and monocytes located in distal colons. (c) Number of goblet cells; these cells were quantified from at least 20 crypts per region in five fields in four different slides per animal. Data are means ± SEM. ^*^ *P* < 0.05, ^**^ *P* < 0.01.

We next analyzed the architecture of the colonic structure and evaluated the histopathology associated with DSS-induced colitis.* T. crassiceps*-infected mice displayed less inflammatory infiltrate as assessed via histological slides (Figures 2(a)-2(b) for BALB/c and Figure 4(b) for C57BL/6). Helminth infection also inhibited the development of cryptitis and neutrophil accumulation within epithelial crypts and in the intestinal mucosa, as uninfected mice that received DSS showed large numbers of neutrophils and macrophages in the injured mucosa of the colon, which correlates directly with clinical disease activity and epithelial injury in colitis (Figure 2(a) upper panel and Figure 2(b)). Moreover,* T. crassiceps*-infected mice and those exposed to DSS had normal numbers of goblet cells as assessed by Alcian blue staining compared to DSS-treated uninfected mice (Figure 2(a) lower panel and Figure 2(c)). The diameter of the colonic submucosa was also measured as a sign of tissue damage and was significantly smaller for the infected group exposed to DSS than for the DSS-treated group (data not shown).

### 3.2. *Taenia crassiceps* Infection Reduces Levels of Systemic Proinflammatory Cytokines during DSS-Induced Colitis

**Figure 3:**
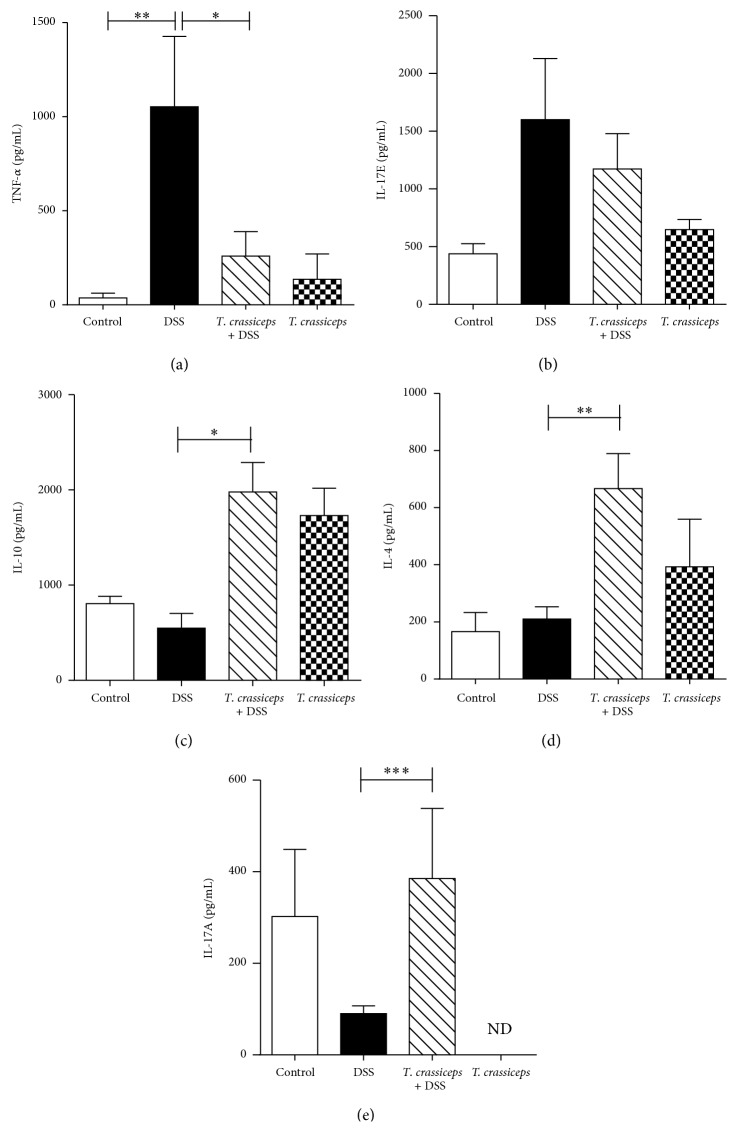
Systemic cytokine profile of* T. crassiceps*-infected and uninfected mice during DSS-induced colitis. (a) Sera TNF-*α* detection. (b) Sera IL-17E detection. (c) Sera IL-10 detection. (d) Sera IL-14 detection. (e) Sera IL-17A detection. Data are means ± SE and are representative of three independent experiments, *n* = 4 mice per group. ^*^ *P* < 0.05 comparing* T. crassiceps*-infected mice and uninfected mice at the end of the experiment.

**Figure 4:**
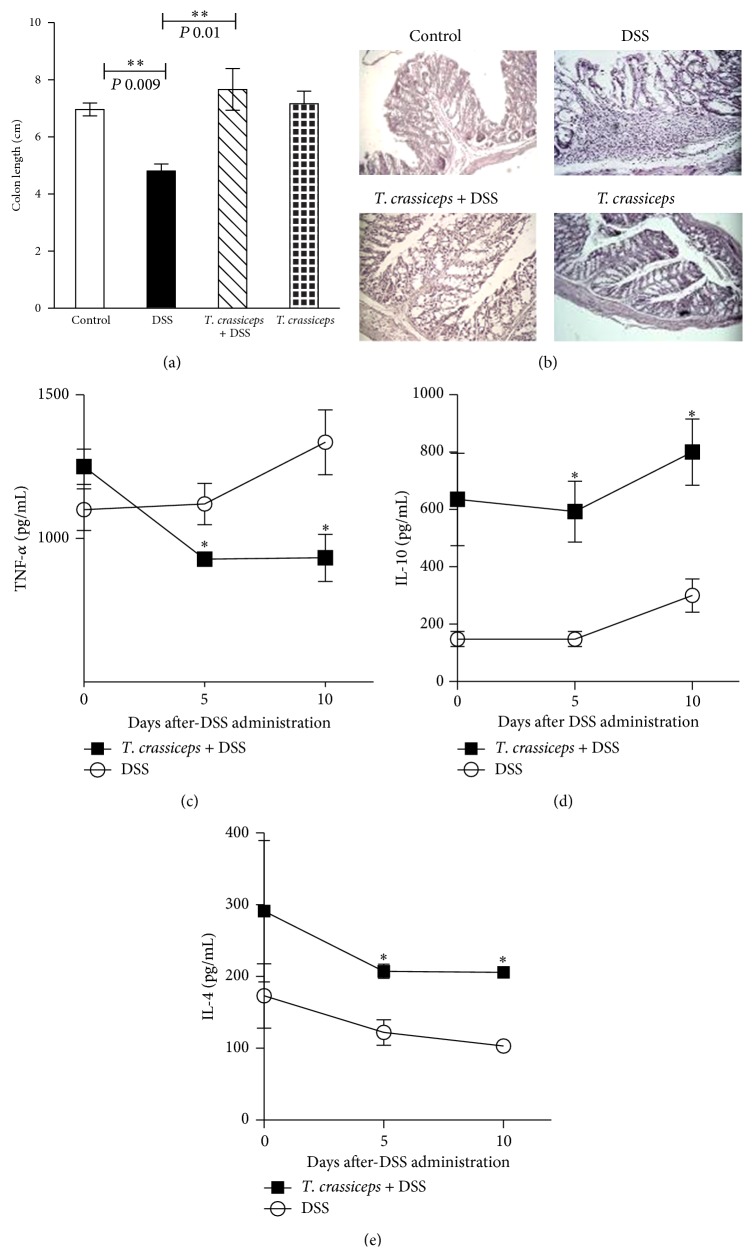
The anticolitic effect of* T. crassiceps* infection is independent of the genetic background of the host. The C57BL/6 mice were infected or not with 40 metacestodes of* T. crassiceps* and ulcerative colitis was induced. (a) Length of the colon for different groups exposed or not exposed to* T. crassiceps* infection. (b) Colon tissue histology stained with H&E and showing colonic inflammation in different groups, magnification = 20x for Control, DSS, and* T. crassiceps* + DSS, 10x for* T. crassiceps*. Serum levels of (c) TNF-*α*, (d) IL-10, and (e) IL-4 detected by ELISA on different days after exposure to DSS. Data are means ± SE and are representative of two independent experiments, *n* = 4 mice per group. ^*^ *P* < 0.05 comparing* T. crassiceps*-infected mice and uninfected mice at the end of the experiment.

Several proinflammatory cytokines as well as inflammatory cells are associated with the severity of IBD [21]. Here we explored whether* T. crassiceps*-infected mice exposed to DSS may modulate systemically the expected increase in proinflammatory cytokines. As shown in Figure 3 for BALB/c mice, DSS-induced colitis in uninfected mice generated an increase in circulating levels of TNF-*α* and IL-17E, two inflammatory cytokines associated with different models of colitis [22]; however,* T. crassiceps*-infected mice displayed lower levels of both cytokines (Figures 3(a)-3(b)). In contrast, infected mice exposed to DSS displayed increased levels of IL-10 and IL-4 compared to uninfected and DSS-treated mice (Figures 3(c)-3(d)). A similar effect was observed in the C57BL/6 strain, in which systemic TNF-*α* levels were downregulated by the presence of* T. crassiceps* infection (Figure 4(c)) but IL-4 and IL-10 levels were significantly elevated (Figures 4(d)–3(e)). Unexpectedly,* T. crassiceps-*infected mice exposed to DSS displayed significant increased levels of IL-17A compared to uninfected and DSS exposed mice (Figure 3(e)).

### 3.3. *Taenia crassiceps* Infection during DSS-Induced Colitis Does Not Modify the Population of T Regulatory Cells

**Figure 5:**
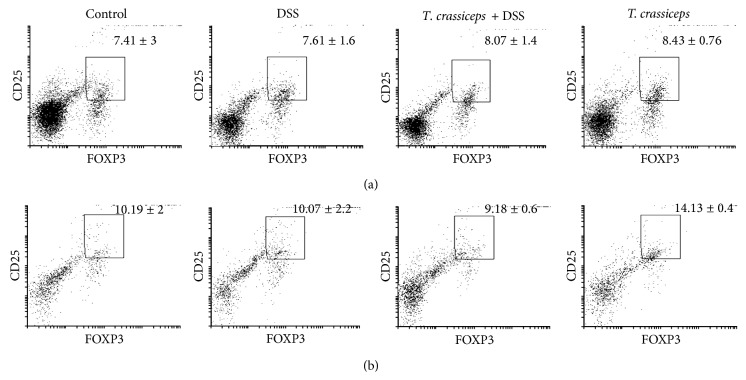
Percentages of T regulatory cells are not altered by* T. crassiceps* infection. Using the Foxp3 reporter mice C.Cg-*Foxp3* ^*tm1Tch*^/J, we analyzed the expression of CD25 and FOXP3 as indicative of the presence of T regulatory cells in (a) spleen cells and (b) peritoneal exudate cells. No significant differences were found among treatments. *n* = 4 mice per group.

Given reports suggesting that the increases in Treg (CD4^+^CD25^+^Foxp3^+^) induced by helminth infections are critically involved in the anticolitic effects of these parasites [23–26], we used a Foxp3 reporter mouse to evaluate the percentages of T regulatory cells in the spleen and peritoneal cavity during* T. crassiceps* infection and DSS-induced colitis. As shown in Figure 5, we did not find significant changes in the percentages of T regulatory cells at either location in infected mice or infected and DSS-treated mice compared to DSS-treated uninfected mice.

### 3.4. *Taenia crassiceps* Infection Reduces Levels of Systemic and Colonic Inflammatory Monocytes but Increases AAMФ Markers in Colonic Tissue during DSS-Induced Colitis

**Figure 6:**
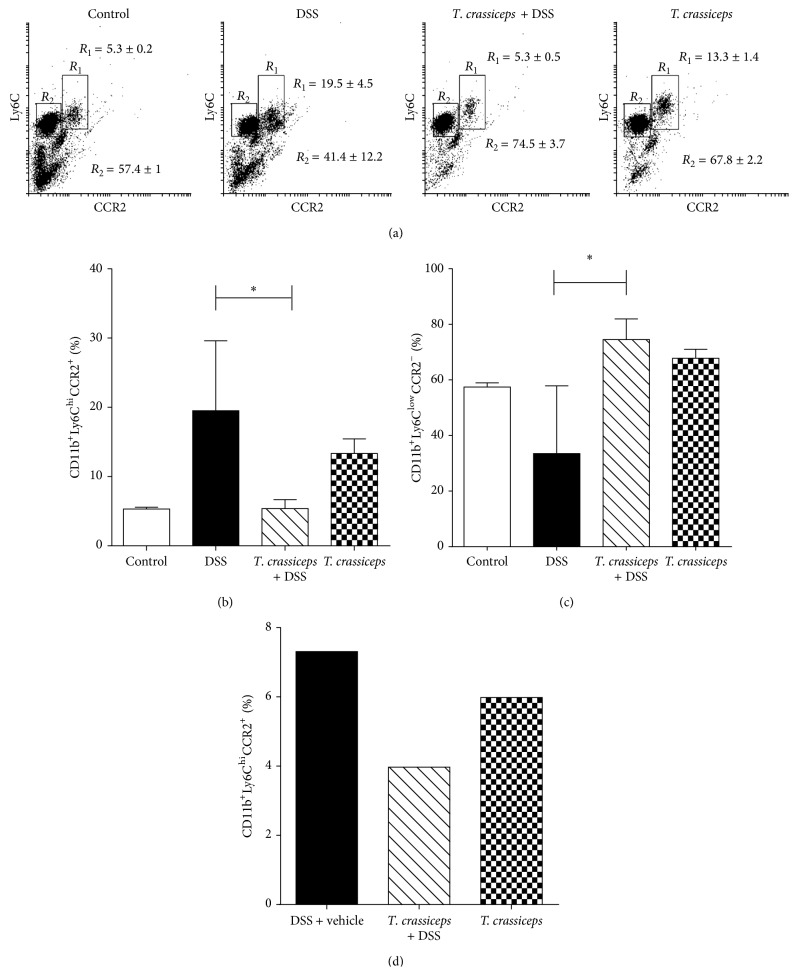
*T. crassiceps* infection reduces the number of inflammatory monocytes during colitis. (a) Representative flow cytometry plots from control mice, DSS-treated mice, and* T. crassiceps* + DSS mice gated on CD11b^+^ living cells isolated from the circulation. Quantification of circulating (b) CD11b^+^Ly6C^hi^CCR2^+^ cells and (c) CD11b^+^Ly6C^lo^CCR2^−^ cells. (d) Percentage of CD11b^+^Ly6C^hi^CCR2^+^ and CD11b^+^Ly6C^lo^CCR2^−^ in cells isolated from the colonic lamina propria. Data are representative of two independent experiments. Values are means ± SE (*n* = 4 mice/group). ^*^ *P* < 0.05, pooled cells for (d).

To examine changes in the recruitment of inflammatory monocytes to the colon tissue during DSS-induced colitis and the effect of* T. crassiceps* infection in these populations we examined circulating levels of CD11b^+^Ly6^hi^CCR2^+^ cells as well as those recruited into the colon. As shown in Figure 6(a), by day 8 after DSS treatment we detected an important systemic increase in the inflammatory monocytes CD11b^+^Ly6^hi^CCR2^+^ in uninfected mice, whereas mice infected with* T. crassiceps* and exposed to DSS displayed a reduced percentage of these inflammatory monocytes (Figures 6(a)-6(b)). In contrast, CD11b^+^Ly6^low^CCR2^−^ cells were increased in these mice (Figure 6(c)).

**Figure 7:**
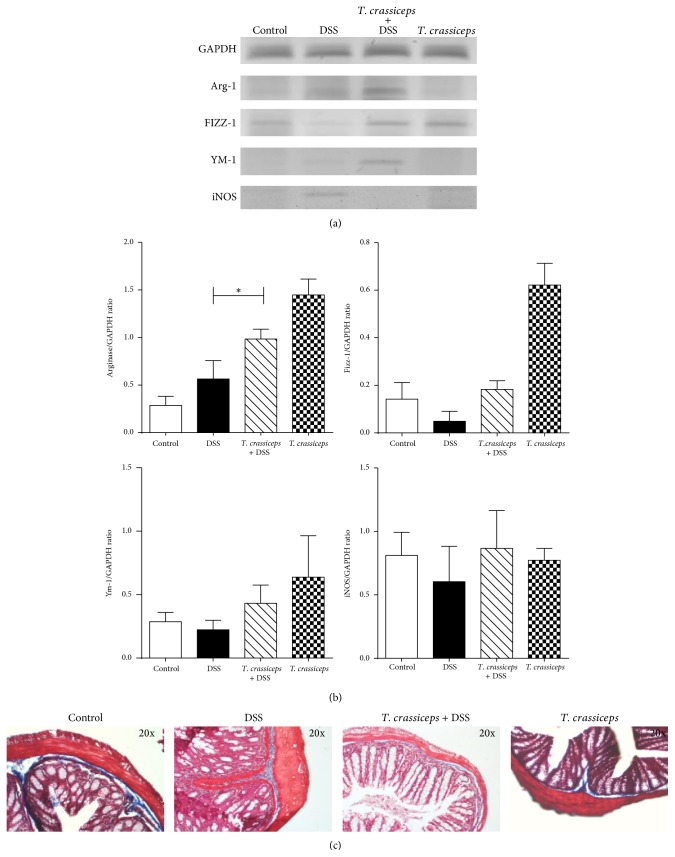
Colon tissue from* T. crassiceps*-infected mice and uninfected mice displays different levels of AAM*Ф*-associated transcripts. (a) Colon tissue was collected at the end of the experiments, and transcript levels of GAPDH, Arginase 1, Fizz1, Ym1, and iNOS were analyzed by RT-PCR. (b) Densitometry of Arg 1, Fizz1, Ym1, and iNOS. (c) Histology with Mason stain for the identification of collagen deposition as a sign of fibrosis.* T. crassiceps* infection does not induce fibrosis during colitis (collagen in dark blue). Magnification = 10x.

To further explore the effect of* T. crassiceps* infection on the development of colitis, we looked for markers of AAM*Ф*s locally in the colon tissue. We found that colons from* T. crassiceps*-infected mice that received DSS positively expressed Arginase 1, FIZZ-1, and Ym-1 (Figure 7(a)), all molecules associated with AAM*Ф*s. In contrast, in the same samples inducible nitric oxide synthase (iNOS) was not detected. It is interesting that uninfected mice exposed to DSS did not express Arginase-1 and Ym-1, but they did express iNOS (Figure 7(a)). Thus, the presence of* T. crassiceps* attenuated the levels of mRNA iNOS, IL-17, and TNF-*α* in both colon tissue and sera.

A classic side effect of strong Th2-type-biased responses induced by helminths is potential development of fibrosis [9, 27]. Here we found that* T. crassiceps*-infected and DSS-treated mice did not display an excess of collagen in the colon tissue, thus ruling out fibrosis as a side effect of this infection during the modulation of colitis (Figure 7(b).

### 3.5. Transfer of AAMФs (F4/80^+^MR^+^) with Suppressive Activity from* T. crassiceps-*Infected Mice Ameliorates DSS-Induced Colitis

**Figure 8:**
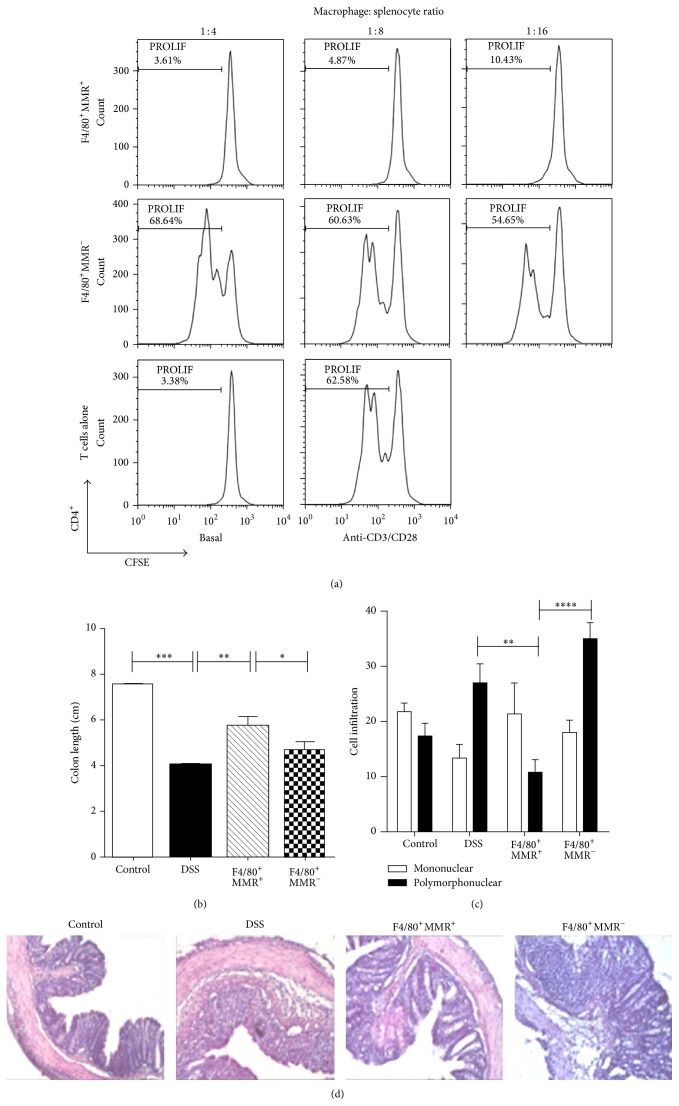
F4/80^+^MR^+^-sorted peritoneal macrophages from* T. crassiceps*-infected mice transferred intraperitoneally into naïve mice inhibit the development of colitis. (a) F4/80^+^MR^+^ peritoneal macrophages sorted from* T. crassiceps*-infected mice inhibit CD4 and CD8 T cell proliferation (data not shown for CD8). In contrast, sorted F4/80^+^MR^−^ macrophages from the same mice do not suppress CD4 cell proliferation. (b) Colon length of mice with ulcerative colitis that received F4/80^+^MR^+^ and F4/80^+^MR^−^ cells. (c) Infiltration of inflammation. (d) Histology of the effect of F4/80^+^MR^+^ adoptive transfer during colitis: magnification is 20x for all the slides shown. Bars represent the mean ± SD from three slides per mouse. ^*^ *P* < 0.05, ^***^ *P* < 0.003, *n* = 5 mice per group. All data are representative of two independent experiments.

Based on our observation that percentages of T regulatory cells were not altered by infection with* T. crassiceps* and because* T. crassiceps* infection recruits AAM*Ф*s into the peritoneal cavity (approximately 35% of peritoneal exudate cells are F4/80^+^MR^+^Arg1^+^) and peritoneal adherent cells suppress T cell proliferation [28], we evaluated whether this population was able to influence the development of DSS-induced colitis. We obtained peritoneal cells from mice previously infected with* T. crassiceps* (6–8 weeks after infection) and sorted them in F4/80^+^MR^+^ (AAM*Ф*s) and F4/80^+^MR^−^ with purity >90% (see Supplemental Figure 1 in Supplementary Material available online at http://dx.doi.org/10.1155/2014/563425). Cells (1 × 10^6^ F4/80^+^MR^+^ or F4/80^+^MR^−^ cells) were adjusted and immediately transferred intraperitoneally to mice previously exposed to DSS. A portion of these purified cells were tested for in vitro suppressive activity on CD4 and CD8 cells from naïve mice. As shown in Figure 8(a), the F4/80^+^MR^+^ cells strongly suppressed the proliferation of CD4 cells as well as CD8 cells (Supplemental Figure 2). In contrast, F4/80^+^MR^−^ cells were unable to inhibit T cell proliferation in response to anti-CD3/CD28 stimuli. It is important to note that the transfer of F4/80^+^MR^+^ cells ameliorated DSS-induced colitis by significantly decreasing bloody diarrhea (Supplemental Figure 3). Moreover, mice that received F4/80^+^MR^+^ cells displayed less signs of colitis, such as shortened colon, and tissue architecture was very well conserved in these mice (Figures 8(b)-8(c)) compared to mice that did not receive cells or mice that received F4/80^+^MR^−^ cells. The transfer of F4/80^+^MR^+^ cells into DSS-treated mice was characterized by much less severe mucosal pathology than in mice that did not receive cells or mice that received F4/80^+^MR^−^ cells, as evidenced by marked destruction of the crypt architecture and a greater influx of inflammatory polymorphonuclear cells, which are largely associated with colitis (Figures 8(c)-8(d)); thus mice receiving F4/80^+^MR^−^ cells showed even worse pathology, with shorter colons and severe signs of cryptitis and loss of colon tissue architecture.

### 3.6. In Vivo Indomethacin Treatment Impairs the Anticolitic Effect of* T. crassiceps* Infection

**Figure 9:**
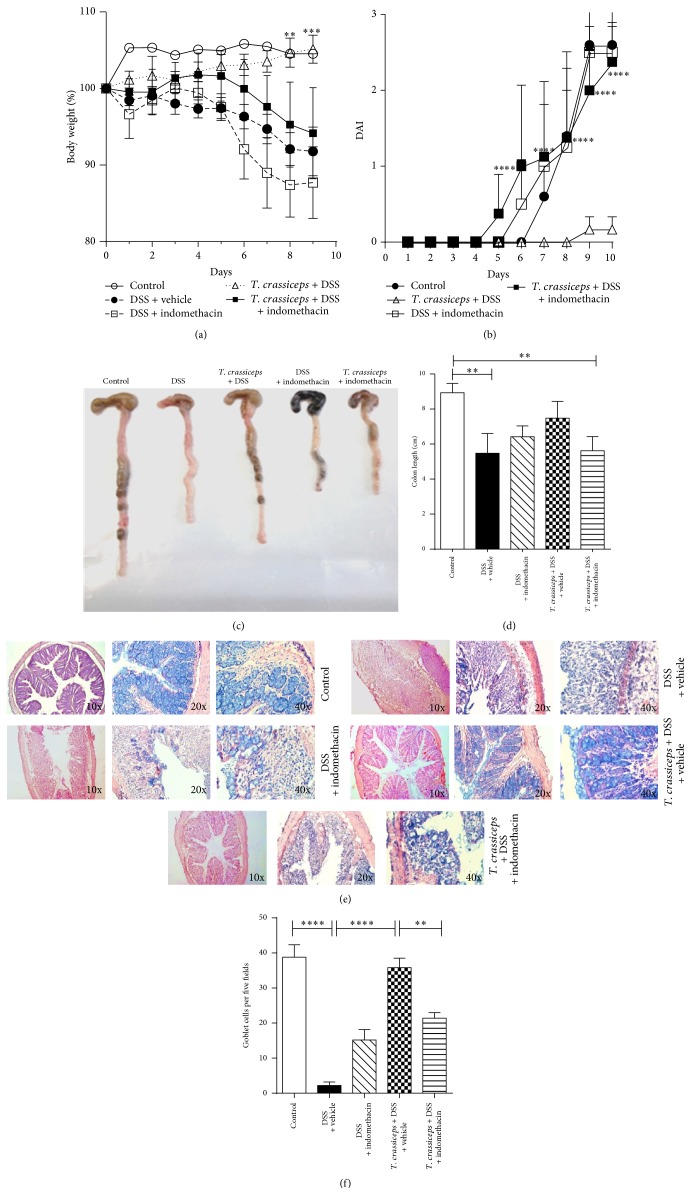
The anticolitic effect of* T. crassiceps* is abrogated by indomethacin treatment. (a) Percentage of weight loss. (b) Disease index. (c) Photograph of gross pathology of colons from different groups of mice. (d) Length of colon in infected and uninfected mice with ulcerative colitis treated or not with indomethacin (3 mg/kg). (e) Histology of colons, left panel colon tissue microphotography (10x) stained with H&E, all other panels are tissue colon stained with Alcian blue to detect goblet cells (20x and 40x, resp.). (f) Number of goblet cells for all the groups. ^*^ *P* < 0.05, ^***^ *P* < 0.003, *n* = 5 mice per group. Similar results were observed in two independent experiments.

Previous work from our lab demonstrated that AAM*Ф*s recruited by* T. crassiceps* infection are strong producers of PGE_2_ [29]. However, PGE_2_ may play a dual role in inflammatory processes, mainly in the gut [30]. Thus, to further elucidate the possible mechanisms involved in the effect of* T. crassiceps* on the amelioration of ulcerative colitis, we injected uninfected and* T. crassiceps*-infected mice with 3 mg/kg indomethacin daily to transiently block PGE_2_ production in vivo. Injections began 2 days before the induction of colitis and were maintained throughout the experiment. Loss of body weight was significantly greater in DSS + indomethacin-treated* T. crassiceps*-infected mice than in DSS-treated* T. crassiceps*-infected mice (Figure 9(a)). This effect correlated with increased signs of morbidity (DAI) over the course of the disease compared to* T. crassiceps*-infected mice similarly treated with DSS (Figure 9(b)). Moreover, increased colon shortening was found in* T. crassiceps*-infected mice exposed to DSS and indomethacin compared to* T. crassiceps*-infected mice exposed to DSS alone (Figures 9(c)-9(d)). It is interesting that indomethacin treatment eliminated the pathological differences between uninfected and* T. crassiceps*-infected mice associated with the development of colitis, as shown in Figures 9(b)–9(e), as a loss of colon tissue architecture was detected even in the presence of this helminth infection. Moreover, a clear change in inflammatory recruitment was found among the different groups of mice. For example, whereas* T. crassiceps* infection reduced the influx of neutrophils into the lamina propria during colitis, uninfected mice recruited higher numbers of neutrophils. What is interesting is that blockage of PGE_2_ by indomethacin treatment significantly increased the influx of neutrophils into the colon of* T. crassiceps-*infected mice (data not shown), and a significant reduction in the number of goblet cells was observed in DSS + indomethacin-treated* T. crassiceps*-infected mice, whereas mice with* T. crassiceps* infection plus colitis retained a higher number of these cells (Figures 9(e)-9(f)). Finally, mice harboring* T. crassiceps* and treated with indomethacin displayed increased production of TNF-*α* and IL-17 compared to* T. crassiceps-*infected mice (data not shown), whereas IL-10 was not affected either at the systemic level or in colonic extracts (data not shown).

## 4. Discussion

The frequency of autoimmune and inflammatory diseases such as multiple sclerosis, type 1 diabetes, and IBD has increased enormously in the past few years, a situation attributed to a lack of exposure to pathogens, especially helminths [4]. Among such inflammatory diseases, IBD has increased at an alarming rate in the past decade, accompanied by improved hygiene, sanitation, and medical conditions and, of course, less infectious diseases, including helminths [4, 11]. Symptoms of IBD are the result of complex interactions among genetic and environmental factors and the immune response [31]. Immunomodulatory effects exerted by helminth parasites on their hosts help to prevent or ameliorate such diseases [14]. Although a large body of evidence indicates that regulatory mechanisms triggered by helminth infections may help to modulate colitis, the precise mechanisms involved are not yet very well understood. Mainly gastrointestinal and transient infections of helminths induce higher levels of Th2 cytokines, induction of T regulatory cells, recruitment and expansion of AAM*Ф*s, and reduction of inflammatory cytokines that results in amelioration in different murine models of colitis [14, 24, 32]. All of these observations suggest that distinct helminths may trigger different pathways to modulate this particular inflammatory disease.

Here we demonstrated that extraintestinal infection with the larval stage of* T. crassiceps* can be added to the growing list of helminth infections with the capacity to modulate colitis; this is just the second cestode reported to induce such protection [14]. Besides the high levels of IL-4 expected with this infection we also found elevated IL-10 levels associated with a downregulation of proinflammatory cytokines. However,* T. crassiceps* infection did not induce higher numbers of T regulatory cells, which is in line with a previous finding on the effect of this infection on experimental autoimmune encephalomyelitis [18]. Therefore, we think that T regulatory cells may play a minor role in our system, even though several authors have found that increased levels of T regulatory cells are associated with improvement in colitis during helminth infections [25, 26]. Here, using T regulatory cell reporter mice we did not find increased levels of T regulatory cells during* T. crassiceps* infection and colitis, but we did observe an improvement in colitis when mice were infected. Based on these observations, our data point to a greater role for AAM*Ф*s (as opposed to regulatory T cells) in the anticolitic effect of* T. crassiceps* infection. Furthermore,* T. crassiceps* infection in DSS-treated mice was characterized by much less severe mucosal pathology than that seen in uninfected control mice, as evidenced by marked destruction of the crypt architecture and an increased influx of inflammatory cells. Furthermore, we demonstrated that in vivo transfer of purified AAM*Ф*s (F4/80^+^MR^+^) recruited for this infection is able to modulate ongoing colitis; thus, it is possible that these cells not only prevent the development of colitis but also may play a curative role. These observations are in line with those recently reported for another cestode,* Hymenolepis diminuta,* which induces AAM*Ф*s associated with the prevention of colitis [9]. However, more studies are needed to confirm whether AAM*Ф*s are the most potent immunoregulatory pathway induced by helminths to reduce colitis.

Neutrophil infiltration is a key event in inflammation of the colon [33, 34]. Here we found that* T. crassiceps* infection during DSS-induced colitis generates a significant change in the populations of cells that infiltrate the colon. We generally observed greater infiltration by monocytes than neutrophils, the latter being the main cell population detected in the absence of this helminth infection. Monocytes can be divided into two subsets: patrolling monocytes that express CD11b^+^CD115^+^CX3CR1^hi^CCR2^lo^Ly6C^lo^ and inflammatory monocytes that express mainly CD11b^+^Ly6C^hi^CCR2^+^ [35]. Inflammatory monocytes accumulate in response to infection or tissue injury, and in most cases they help to clear pathogens [36]. However, in some pathology and especially in IBD, the recruitment of inflammatory monocytes into damaged tissue frequently worsens the inflammation [35]. The recruitment of inflammatory Ly6C^hi^ monocytes into adult mucosa is dependent on CCR2 expression [37]. Here we found that the circulating levels and recruitment of CD11b^+^Ly6C^hi^CCR2^+^ inflammatory monocytes into the colon were significantly reduced by the presence of* T. crassiceps* infection; in contrast, CD11b^+^Ly6C^lo^CCR2^−^ cells increased during infection. This is the first time that it has been reported that an extraintestinal helminth infection is able to modulate these cell populations. Such modulation may have an impact on the development of colitis and also may favor development into AAM*Ф*s, as demonstrated by the expression of Arg 1, FIZZ 1, and YM1 in colon tissue. Thus, a reduction in the recruitment of inflammatory monocytes together with an increase in AAM*Ф*s in the colon may be a strong anticolitic mechanism triggered by* T. crassiceps* infection.

An interesting finding is that indomethacin treatment to inhibit PGE_2_ production in vivo during* T. crassiceps* infection and exposure to DSS completely abrogated the anticolitic effect of this infection. This result suggests that the in vivo capacity of* T. crassiceps* infection to suppress DSS-induced colitis is highly dependent on the ability of the host to produce PGE_2_, maybe in response to commensal or parasite-derived stimulation. These findings are in line with those reported by Bao et al. [38], who found that PGE_2_ plays a fundamental role in regulating the immune response to colitis as well as modulating Th1/Th2 responses [38]. Specifically, when we blocked in vivo PGE_2_ using indomethacin, the anticolitic effect of* T. crassiceps* infection was clearly abrogated, but IL-10 levels still remained elevated, indicating a major role for PGE_2_ during* T. crassiceps*-mediated amelioration of colitis. In line with these data, other authors have found that even in the absence of IL-10, colitis can be modulated [39]. As further support of this idea, our group previously reported that macrophages obtained from* T. crassiceps-*infected mice are able to produce significantly elevated levels of PGE_2_ in response to stimulation with LPS [29], a molecule to which epithelial and intestinal macrophages are highly exposed.

Moreover, we found the number and size of goblet cells increased in infected animals with DSS-induced colitis compare with colitic mice, suggesting that* T. crassiceps*, like other helminths, can help in preserving these cells [40, 41]. Goblet cells are involved in regulating both the mucosal barrier and the relative composition of the luminal microbiota by mucin production [42]. The high expression of IL-4 in* T. crassiceps* + DSS mice suggests that this infection may maintain the numbers of goblet cells. The production of mucus by these cells could limit bacterial access to epithelial cells and prevent chronic inflammation [43]. Thus, an increase or recruitment of AAM*Ф*s in the lamina propria seems to be necessary for anti-inflammatory activity. We demonstrated that* T. crassiceps* infection during colitis is able to promote the polarization of AAM*Ф*s, thereby attenuating the expression of inflammatory cytokines, preventing damage to the colon and the development of colitis. The relationship between infiltrating AAM*Ф*s and prognosis in colitis has not been analyzed; therefore, the distribution and function of macrophages in experimental ulcerative colitis need to be evaluated further. Another interesting finding here is a trend to reduce IL-17E by* T. crassiceps* infection in colitis, but surprisingly IL-17A levels were increased in the same group; in line with these data are several findings indicating that the presence of IL-17A has an anticolitic effect, given that IL-17AKO mice became dramatically susceptible to DSS-induced colitis [44] and other researchers reported similar findings in distinct models of colitis, these data are in this moment very difficult to explain, and suggest that Th17 family has complex functions during different inflammatory diseases [45]. The mechanisms regarding the differential IL-17 modulation by* T. crassiceps* infection remain to be elucidated. Finally, the modulation of Th1- and Th17-type cytokines observed here accords with Tao et al. [22], who suggested that inactivation of STAT1 and STAT3 may contribute to resolving different models of colitis. We recently found that infection with* T. crassiceps* or its excreted/secreted products are able to inhibit STAT1 phosphorylation in macrophages and splenocytes in response to IFN-*γ* [46]. Thus, it appears that multiple mechanisms can be triggered by* T. crassiceps* infection or its products to modulate inflammatory responses.

## 5. Conclusion

We found that extraintestinal infection with* T. crassiceps* significantly reduced both symptoms and colonic inflammation associated with ulcerative colitis independently of the genetic background. Moreover, AAM*Ф*s and prostaglandins may play a critical role in avoiding colonic inflammation and perhaps inhibiting recruitment of inflammatory monocytes CD11b^+^Ly6C^+^CCR2^+^ into the lamina propria of the colon.
